# One Health Approaches to Trace *Mycobacterium leprae*’s Zoonotic Potential Through Time

**DOI:** 10.3389/fmicb.2021.762263

**Published:** 2021-10-21

**Authors:** Christian Urban, Alette A. Blom, Saskia Pfrengle, Kathleen Walker-Meikle, Anne C. Stone, Sarah A. Inskip, Verena J. Schuenemann

**Affiliations:** ^1^Institute of Evolutionary Medicine, University of Zurich, Zurich, Switzerland; ^2^Department of Archaeology, University of Cambridge, Cambridge, United Kingdom; ^3^Department of History, King’s College London, London, United Kingdom; ^4^School of Human Evolution and Social Change, Arizona State University, Tempe, AZ, United States; ^5^School of Archaeology and Ancient History, University of Leicester, Leicester, United Kingdom

**Keywords:** *Mycobacterium leprae*, leprosy, palaeomicrobiology, zoonosis, One Health, palaeopathology, ancient biomolecules, ancient pathogens

## Abstract

Hansen’s disease (leprosy), mainly caused by infection with *Mycobacterium leprae*, has accompanied humanity for thousands of years. Although currently rare in Europe, there are over 200,000 new infections annually in South East Asia, Africa, and South America. Over the years many disciplines – palaeopathology, ancient DNA and other ancient biomolecules, and history – have contributed to a better understanding of leprosy’s past, in particular its history in medieval Europe. We discuss their contributions and potential, especially in relation to the role of inter-species transmission, an unexplored phenomenon in the disease’s history. Here, we explore the potential of interdisciplinary approaches that understand disease as a biosocial phenomenon, which is a product of both infection with *M. leprae* and social behaviours that facilitate transmission and spread. Genetic evidence of *M. leprae* isolated from archaeological remains combined with systematic zooarchaeological and historical analysis would not only identify when and in what direction transmission occurred, but also key social behaviours and motivations that brought species together. In our opinion, this combination is crucial to understand the disease’s zoonotic past and current potential.

## Introduction

Hansen’s disease (HD) – colloquially known as leprosy – is a chronic infectious disease whose main causative agent is *Mycobacterium leprae* (Han et al., 2008). Leprosy is considered eliminated in most countries, yet over 200,000 new cases are reported annually (WHO, 2020). At the genetic level some *M. leprae* strains are highly localised geographically, while others are more widely spread (Benjak et al., 2018; Avanzi et al., 2020). Although originally believed to only affect humans, research increasingly shows that many species are affected; *M. leprae* was first identified in a non-human species in the United States, in nine-banded armadillos (*Dasypus novemcinctus*, e.g., Folse and Smith, 1983). Recently, *M. leprae* was found in modern Eurasian red squirrel (*Sciurus vulgaris*) from Brownsea Island, southern England (Avanzi et al., 2016) and in various non-human primates (Honap et al., 2018). Moreover, current research highlights ticks (Ferreira et al., 2018) and amoebae (Lahiri and Krahenbuhl, 2008; Wheat et al., 2014) as potential vectors or reservoirs for *M. leprae*, adding further complexity to the disease’s transmission. Considering evidence for the long evolutionary history of *M. leprae* as an obligate pathogen characterised by a downsized genome and its estimated 13.9 MYA divergence from its closest relative, *M. lepromatosis*, it is evident that humans have not been the only host in the past (Dagan et al., 2005; Gómez-Valero et al., 2007; Singh et al., 2015). Exactly which animal species have been affected throughout history and when, however is unstudied.

**FIGURE 1:**
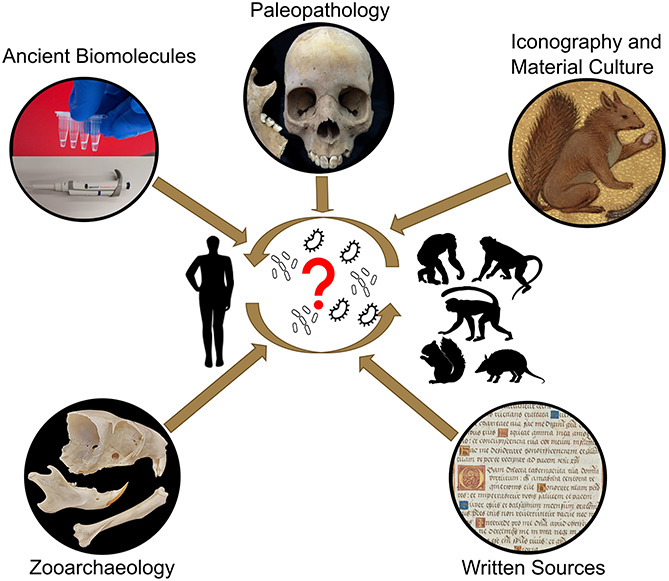
Schematic illustration of the key questions regarding the interspecies transmission of *M. leprae* and potential disciplines to solve open questions.

A better comprehension of the natural reservoirs and animal hosts of *M. leprae* is key to revealing when and in which direction the bacterium transmitted between humans and animals, and which different animals may have been hosts. This knowledge can help us interpret historic declines and lingering reservoirs and HD pockets today, potentially explaining the disease’s persistence despite significant eradication efforts. Catalysts for interspecies transmissions are, however, complex, and often linked to changes in lifestyle including population growth, mobility, culture, companion animals, urbanisation, animal breeding, trade, or changes in land usage (Daszak et al., 2001; Karesh et al., 2012; Jones et al., 2013). To fully address the question of the origin of *M. leprae*, an integrated approach is necessary. This paper describes knowledge that has been and can be obtained from multiple disciplines (palaeopathology, zooarchaeology, ancient biomolecules, and history) and how we propose to combine these to study *M. leprae’s* origin, evolution, and interspecies transmissions in human and animal populations (Figure 1).

## Palaeopathology

Palaeopathology is the study of health and disease in the past through the analysis of archaeological skeletal remains. Leprosy has long been of interest to palaeopathologists as it can leave a combination of highly distinctive skeletal lesions: the nerve damage caused by *M. leprae* invasion can lead to direct and indirect bone changes in the hands, feet, and facial bones (Table 1; Roberts and Buikstra, 2019). This field has been pivotal in showing that *M. leprae* has likely infected humans for at least 5,500 years (Köhler et al., 2017). Combined with historic sources, palaeopathology has shown that HD afflicted individuals of different social standings, background and sex, and that responses to the afflicted were not always negative, challenging modern perceptions of the ostracised medieval “leper” (Roberts, 2020; Brenner and Touati, 2021). Geospatial analyses of past cases have highlighted important sociocultural factors in the disease’s spread including trade, military conflict, and possibly pilgrimage (Roffey et al., 2017; Roberts, 2020; Touati, 2021). However, in understanding past case distribution, the trade in, keeping of and living with animals, which may play a crucial role in the spread and persistence of (infectious) disease within human society, has not yet been considered. Including animals in such narratives and extending palaeopathological approaches to zooarchaeology provides significant potential to assess this important and overlooked facet of HD’s history.

**TABLE 1:** Comparison of human and Eurasian red squirrel (*Sciurus vulgaris*) pathology caused by *M. leprae* infection.

	Humans	*Sciurus vulgaris*
Macroscopic lesions	Patches of hypopigmented or red skin; Swelling and thickening of limbs, forehead, brows, and earlobes; Nodular thickening of the nose; Nodular and cutaneous lesions; Alopecia.	Swelling of nose, lips, eyelids, ears, and extremities; Crusty skin thickening of nose, lips, eyelids, ears, and extremities; Keratinisation of ear pinna; Wart-like/cutaneous growths on nasal area, ears, and extremities; Alopecia.
Histopathological alteration	Epithelioid granulomas; Lymphocytes and histiocytes in irregular, unorganised, or linear clusters; Sheet of foamy macrophages (Scollard, 2016); High number of acid-fast bacilli.	Granulomatous dermatitis; Sheets of epithelioid macrophages; High number of acid-fast bacilli.
Neurological manifestations	Acid-fast bacilli in nerves; Swelling of (peripheral) nerves; Neuritis.	Acid-fast bacilli in nerves; Swelling of the (peripheral) nerves; Neuritis.
Skeletal manifestation	Resorption of the nasal spine; Increased porosity, roundening, and thickening of the nasal aperture, with possible enlargement or narrowing of the opening; Increased porosity and resorption of internal nasal bones; Increased porosity in, thinning of, and new porous bone on the oral and nasal surface of the palate; Increased porosity, lytic lesions, and porous bone growth on the ear ossicles; New bone growth and resorption of finger and foot bones; Dense and porous bone deposits on the fibulae and tibiae, most often along the interosseous crest; Volar grooving on finger bones due to flexing.	*Increased porosity, roundening and thickening of the nasal aperture, with possible enlargement or narrowing of the opening; Increased porosity and resorption of internal nasal bones; Increased porosity in, thinning of, and new porous bone on the oral and nasal surface of the palate; Resorption of the maxillary alveolar bone; Increased porosity, lytic lesions, and porous bone growth on the ear ossicles; New bone growth and resorption of finger and foot bones; Dense and porous bone deposits on the fibulae and tibiae, most often along the interosseous crest.*

**Cursive print* presents theorised/expected pathology. *S. vulgaris* pathology based on Avanzi et al. (2016).*

## Zooarchaeology

Zooarchaeology studies the remains of past animals and our interactions with them. Such research has revolutionised our understanding of human behaviour, lifestyle, diet, and society (O’Connor and O’Connor, 2008). Although far less common in zooarchaeology, palaeopathological studies on animal remains have been key for highlighting changes in animal management practices, shifts in human subsistence strategies and changing attitudes toward animals (Thomas, 2019). Furthermore, it has been possible to recover and identify pathogen DNA from animal bones, including *Y. pestis* from a rat (Morozova et al., 2020) showing the potential to recover disease-causing organisms. There thus exists a significant opportunity to assess mammal remains that are potentially capable of hosting *M. leprae* in the past, including Eurasian red squirrels (*Sciurus vulgaris*), with a view to identifying past cases. While rarely complete, red squirrel bones have been found at many sites across Britain. Consideration of the time period, context of the assemblage and type of skeletal elements present, can provide clues as to the nature of interactions taking place between humans and animals.

Assessment of leprosy in zooarchaeology is prevented by the lack of research into whether potential reservoir animals can develop skeletal changes that would be recognisable. It is often assumed that (small) animals would not live long enough for skeletal changes of disease to develop (Bartosiewicz, 2016). However, higher body surface area to volume ratio in small mammals causes a higher metabolic rate, potentially leading to faster disease progression. Lower skin temperature at the extremities forms a unique environment for *M. leprae* to replicate, as in armadillos. This, combined with the thin skin that covers the extremities, would not require advanced granulomas or pyogenic infection to affect bone, especially in the ears, nose, hands, and feet. Evidence for this argument can be gleaned from veterinary studies of red squirrels and armadillos that have tested positive for *M. leprae* (e.g., Sharma et al., 2013; Avanzi et al., 2016), in which skin lesions are similar to those found in humans (Table 1).

## Ancient Biomolecules

In contrast to palaeopathological investigations, research on ancient biomolecules can provide direct evidence for the presence of a particular pathogen such as *M. leprae* by either detecting its DNA (Schuenemann et al., 2013, 2018) or specific lipids – mycolic acids – of its cell wall in ancient human remains (Minnikin et al., 2011; Inskip et al., 2015). The links between host immunity and HD are observed in past populations through analyses of genes (Krause-Kyora et al., 2018) and peptides linked to human immune genes in ancient individuals tested positive for *M. leprae* DNA (Fotakis et al., 2020). Although most current studies focus on ancient DNA (aDNA), the combination of different ancient biomolecules – multi-omics investigations – can be seen as a clear future direction in ancient pathogen research, which may enable the detection of active infections in the past through the simultaneous presence of ancient pathogen and host immune peptides.

The first medieval *M. leprae* genomes reconstructed from ancient human remains were among the first reconstructed ancient pathogen genomes in the palaeogenetics field (Schuenemann et al., 2013) and showcase the potential of aDNA to reveal past and present genome variation, genetic diversity, and genomic structure of *M. leprae*. Together with several follow up studies (Mendum et al., 2014; Schuenemann et al., 2018; Fotakis et al., 2020; Neukamm et al., 2020) a total of 19 high-coverage ancient *M. leprae* genomes have been published to date, uncovering two significant characteristics of the bacterium: a genetic continuity over the last 1000 years (Schuenemann et al., 2013) and a high genetic diversity across northwestern medieval Europe (Schuenemann et al., 2018). The evolutionary relationships of the ancient and modern genomes can be visualised in phylogenetic trees (Pavlopoulos et al., 2010). These branching diagrams allow tracing of the divergence of *M. leprae* strains from their most recent common ancestor into eight main branches with genetic similarities (Schuenemann et al., 2018). Specifically, *M. leprae* strains of branch 0, 2F, 3, and 4 were prevalent in ancient Europe (Schuenemann et al., 2018) representing almost the entire modern diversity of *M. leprae* strains. These results call into question previous hypotheses of the disease’s origin being in India or Northeast Africa (Monot et al., 2005; Robbins et al., 2009). In addition, they also invite, further investigations of the diversity of strains in areas where leprosy persists and how this relates to previous population movements.

Furthermore, medieval *M. leprae* genomes have contributed to our understanding of the diversity and distribution of strains in animal reservoirs. Many ancient *M. leprae* genomes from humans show a close genetic relationship to strains isolated from modern animal reservoirs including the nine-banded armadillo (Truman et al., 2011), British red squirrels (Avanzi et al., 2016), and non-human primates (Honap et al., 2018). The first connection was found on branch 3 between modern *M. leprae* strains isolated from armadillos (Truman et al., 2011) and late medieval strains from northern Europe located basally in the phylogenetic tree (Schuenemann et al., 2013). This finding suggests a European origin for leprosy in the Americas with European settlers introducing the disease, which was subsequently transmitted to armadillos (Schuenemann et al., 2013). Today, zoonotic transmission of HD from armadillos to humans is one of the driving forces of HD infection in the Americas (e.g., Oliveira et al., 2019). Further phylogenetic analyses revealed a close relationship between seven genomes recovered from medieval human burials in the United Kingdom and Denmark (Schuenemann et al., 2018) and modern *M. leprae* strains from British red squirrel (Avanzi et al., 2016) on branch 3. Squirrels therefore might have had a similar influence on the spread of the disease in Europe in the past. Outside branch 3, medieval European strains can be linked to modern ones isolated from non-human primates on branch 4 and branch 0. In particular, an early medieval *M. leprae* genome from the Czech Republic is basal to almost the entire branch 4 including to those genomes isolated from modern chimpanzees and sooty mangabeys (Honap et al., 2018; Schuenemann et al., 2018). In addition, the branch 0 *M. leprae* genome isolated from a modern Asian macaque is related to medieval European genomes from the United Kingdom and Denmark (Honap et al., 2018; Schuenemann et al., 2018). Overall, these findings across different branches in the phylogeny reveal several interspecies transmission events across time. However, the direction of these events – zoonotic or anthroponotic – cannot yet be deciphered. To elucidate such transmission events, it is essential to identify both modern and ancient animal reservoirs and the opportunities for interactions among species.

## Historical Sources on Animal Reservoirs

Analysis of historical sources can help identify opportunities for transmission in the past, although we have not found evidence of animals attested as reservoirs of *M. leprae* in the historic record. Armadillos are hunted for their meat in many areas in North, Central, and South America, and recent work in Pará, Brazil, shows a link between regular consumption of armadillo meat and the presence of leprosy antibodies (da Silva et al., 2018). Apart from their meat, armadillos are also handled extensively in many traditional crafts, such as the manufacture of charangos (a small stringed instrument). The consumption of armadillo flesh is longstanding; in the sixteenth century Gonzalo Fernández de Oviedo (1478–1557) commented on the tastiness of their flesh (De Oviedo, 2011) while Francisco Hernández (1514–1587) recorded that armadillos were hunted for their meat, and their tails and shells were widely used, even suggesting that the ground tail was a useful remedy for syphilis, curing a “New World” disease with a “New World” animal (Hernádez, 2001). However, it has not been possible to pinpoint when the transmission of leprosy from humans to armadillo took place, although it likely would have been multiple events, owing to the huge geographic distance between species of infected armadillos and the genetic differences between the identified strains (Truman et al., 2011; Sharma et al., 2015).

Regarding potential avenues for transmigration of *M. leprae* between red squirrels and humans in the past, we have extensive historic evidence for close contact between the two species in the medieval period, through fur trade, pet keeping, and meat consumption. Squirrel fur was the most popular fur in the High and Late Middle Ages in Western Europe. It was used to both line garments and served as decorative edgings on collars, cuffs, hems, tippets, and *fitchets* (slits in robes for hands) (Ewing, 1981; Newton, 1999). For England alone, red squirrel fur was imported in huge quantities from Scandinavia and Russia. Novgorod’s entire economy was based on the squirrel fur trade, and it only declined at the end of the fifteenth century (Martin, 1986). The quantities of squirrel skins used were vast; e.g., 79,220 trimmed white belly skins (*miniver*) were purchased for the English royal household in the year 1344–1345 alone (Veale, 1966). These wares would be sold at fairs or by merchants, and prepared for sale by skinners, the latter living in most towns. Large quantities of squirrel skins were also imported from Ireland, Scotland, Spain, Poland, Hungary, and Southern Italy. There may have been a small-scale trade of locally trapped English squirrel fur in the period, but it would have been in small quantities compared to the vast amount of imported squirrel fur.

Both squirrels and their fur appear frequently in accounts, iconography, material culture, and other sources. Apart from the fur trade, people in medieval England lived in the presence of a large population of red squirrels. Aside from observing them in trees or wearing them on their clothes, squirrels were also popular pets. Kept with collars and leashes, taking one’s squirrel for a walk was not an eccentric practice but widespread, particularly among elite women, both secular and in religious orders (Walker-Meikle, 2012).

## Research Limitations

A clear understanding of the natural reservoirs and occasional hosts of *M. leprae* is currently lacking, possibly in part due to the laborious task of integrating data from different fields into one usable database (Driscoll et al., 2011). Humans are not the only species affected by leprosy, and perhaps not even the primary host, either today or in the past. Data from ancient biomolecules, in particular from aDNA, will be important for understanding the transmission among human and animal hosts. Historic and genetic research suggest medieval squirrels may have been infected and affected by leprosy. Likewise, genetic evidence of *M. leprae* exchange with non-human primates points to multiple jumps, with strains in African and Asian primates mostly similar to those found in nearby human populations (Honap et al., 2018; Hockings et al., 2020). One exception is a strain found in a chimpanzee in Côte d’Ivoire that clustered in branch 2F (Hockings et al., 2020). Strains in this branch have been found in medieval Europe and modern Ethiopia but not in West Africa (Benjak et al., 2018; Schuenemann et al., 2018). While there is evidence of pathogen exchange among non-human primates and humans (e.g., Sharp and Hahn, 2011; Devaux et al., 2019), non-human primate habitats are largely in environments that are not conducive to aDNA preservation and there is a lack of historical data prior to the last few centuries. Such multidisciplinary approaches would benefit from additional research on the modern *M. leprae*-complex to understand the role of other members of this group in disease causation in the past and today (Ploemacher et al., 2020).

Studying insect vectors to understand *how* leprosy jumped from one species to another remains a viable research direction, but insect remains are mainly inaccessible in archaeological contexts, and aDNA retrieval is highly challenging (Reiss, 2006). More promising, is the analysis of zooarchaeological remains. Such research is limited by the lack of palaeopathological work on animal bones and the scarce reporting of squirrel bones from the archaeological record. At present, no skeletal remains of squirrels (or other animals) with pathological lesions consistent with HD have been reported, although this is not to say they do not exist. This is particularly true in leprosy-endemic areas in Africa and Asia. In addition, aDNA research is still dependent on the amount of material that can be extracted from a sample and the DNA preservation per individual sample. The former is more limited in small mammal skeletons, such as squirrels, compared to human remains, while the latter is highly affected by the hot and moist climate in tropical regions (Bollongino et al., 2008; Hofreiter et al., 2015).

Overall, palaeopathological investigation of zooarchaeological remains, combined with ancient biomolecular investigations, including aDNA, ancient lipids, and proteomics, as well as examining the host genetics are important to address questions about past intra- and interspecies transmission of *M. leprae*.

## Outlook: How Studies on Leprosy’s Past Can Contribute to One Health Approaches

In revealing HD’s zoonotic past, we can illustrate the importance of considering animals as reservoirs for the disease’s long-term history and the need to address social practices and attitudes toward animals which are conducive to its spread. Furthermore, it can contribute to a better understanding of historic declines such as the decrease of HD in 16th-century Europe, which remains unresolved (Demaitre, 2007).

• Examination of modern squirrels and armadillos with HD can help us identify skeletal lesions associated with leprosy in non-human animals. This can be used to identify cases in archaeological animals.
• This knowledge can then be used to study human and animal remains from the same site or sites close together, to identify potential locations and times for transmission, and identify samples for genetic analysis.
• To understand why transmissions took place at specific times or where there is potential for transmission, historical and archaeological analysis should be undertaken to identify key social behaviours and motivations that brought species together.
• Genetic evidence of *M. leprae* isolated from archaeological remains of humans and animals, in combination with data from other archaeological and modern cases, can be used to qualify the relationship between human and animal strains and to identify when and in which direction transmission occurred.

Overall, an interdisciplinary approach (Figure 1) is necessary; one that understands disease as a biosocial phenomenon – which is a product of both infection with *M. leprae* and social behaviours that facilitate transmission and spread. Such approaches have the potential to help us understand key factors behind zoonotic transmission across time and can therefore contribute valuable knowledge for the eradication of the disease today.

## Author Contributions

VS, SI, AS, and KW-M conceived the article and research questions. CU, AB, SP, and KW-M reviewed the literature and together with VS, SI, and AS they summarized and interpreted the data. All authors wrote the manuscript together and approved the final version.

## Conflict of Interest

The authors declare that the research was conducted in the absence of any commercial or financial relationships that could be construed as a potential conflict of interest.

## Publisher’s Note

All claims expressed in this article are solely those of the authors and do not necessarily represent those of their affiliated organizations, or those of the publisher, the editors and the reviewers. Any product that may be evaluated in this article, or claim that may be made by its manufacturer, is not guaranteed or endorsed by the publisher.

## References

[B1] Avanzi C. Del-Pozo J. Benjak A. Stevenson K. Simpson V. R. Busso P. (2016). Red squirrels in the British Isles are infected with leprosy bacilli. *Science* 354 744–747. 10.1126/science.aah3783 27846605

[B2] Avanzi C. Singh P. Truman R. W. Suffys P. N. (2020). Molecular epidemiology of leprosy: an update. *Infect. Genet. Evol.* 86:104581. 10.1016/j.meegid.2020.104581 33022427

[B3] Bartosiewicz L. (2016). The palaeopathology of wild mammals in archaeology = Vadon élõ emlõsállatok betegségei a régészetben. *Archeometriai Mûhely* 13 19–30. 10.2307/j.ctvh1djdq.8

[B4] Benjak A. Avanzi C. Singh P. Loiseau C. Girma S. Busso P. (2018). Phylogenomics and antimicrobial resistance of the leprosy bacillus *Mycobacterium leprae*. *Nat. Commun.* 9:352. 10.1038/s41467-017-02576-z PMC5783932 29367657

[B5] Bollongino R. Tresset A. Vigne J.-D. (2008). Environment and excavation: pre-lab impacts on ancient DNA analyses. *C. R. Palevol.* 7 91–98. 10.1016/j.crpv.2008.02.002

[B6] Brenner E. Touati F.-O. (2021). “Leprosy and Identity in the Middle Ages,” in *From England to the Mediterranean*, eds Brenner E. Touati F. O. (Manchester: Manchester University Press).

[B7] da Silva M. B. Portela J. M. Li W. Jackson M. Gonzalez-Juarrero M. Hidalgo A. S. (2018). Evidence of zoonotic leprosy in Pará, Brazilian Amazon, and risks associated with human contact or consumption of armadillos. *PLoS Negl. Trop. Dis.* 12:e0006532. 10.1371/journal.pntd.0006532 29953440 PMC6023134

[B8] Dagan T. Blekhman R. Graur D. (2005). The “Domino Theory” of gene death: gradual and mass gene extinction events in three lineages of obligate symbiotic bacterial pathogens. *Mol. Biol. Evol.* 23 310–316. 10.1093/molbev/msj036 16237210

[B9] Daszak P. Cunningham A. A. Hyatt A. D. (2001). Anthropogenic environmental change and the emergence of infectious diseases in wildlife. *Acta Trop.* 78 103–116. 10.1016/s0001-706x(00)00179-0 11230820

[B10] De Oviedo G. F. (2011). “Índice De Ilustraciones,” in *Sumario de la Natural Historia de las Indias*,ed. De Oviedo G. F. (Frankfurt: Vervuert Verlagsgesellschaft), 365–368.

[B11] Demaitre L. (2007). *Leprosy in Premodern Medicine: A Malady of the Whole Body.* Baltimore, MD: JHU Press.

[B12] Devaux C. A. Mediannikov O. Medkour H. Raoult D. (2019). infectious disease risk across the growing human-non human primate interface: a review of the evidence. *Front. Public Health* 7:305. 10.3389/fpubh.2019.00305 31828053 PMC6849485

[B13] Driscoll T. Gabbard J. L. Mao C. Dalay O. Shukla M. Freifeld C. C. (2011). Integration and visualization of host–pathogen data related to infectious diseases. *Bioinformatics* 27 2279–2287. 10.1093/bioinformatics/btr391 21712250 PMC3150046

[B14] Ewing E. (1981). *Fur in Dress*, 1st Edn. London: Batsford.

[B15] Ferreira J. da S. Souza Oliveira D. A. Santos J. P. Ribeiro C. C. D. U. Baêta B. A. (2018). Ticks as potential vectors of Mycobacterium leprae: use of tick cell lines to culture the bacilli and generate transgenic strains. *PLoS Negl. Trop. Dis.* 12:e0007001. 10.1371/journal.pntd.0007001 30566440 PMC6326517

[B16] Folse D. S. Smith J. H. (1983). Leprosy in wild armadillos (*Dasypus novemcinctus*) on the Texas Gulf Coast: anatomic pathology. *J. Reticuloendothel. Soc.* 34 341–357. 6644691

[B17] Fotakis A. K. Denham S. D. Mackie M. Orbegozo M. I. Mylopotamitaki D. Gopalakrishnan S. (2020). Multi-omic detection of Mycobacterium leprae in archaeological human dental calculus. *Philos. Trans. R. Soc. Lond. B Biol. Sci.* 375:20190584. 10.1098/rstb.2019.0584 33012227 PMC7702802

[B18] Gómez-Valero L. Rocha E. P. C. Latorre A. Silva F. J. (2007). Reconstructing the ancestor of Mycobacterium leprae: the dynamics of gene loss and genome reduction. *Genome Res.* 17 1178–1185. 10.1101/gr.6360207 17623808 PMC1933519

[B19] Han X. Y. Seo Y.-H. Sizer K. C. Schoberle T. May G. S. Spencer J. S. (2008). A new Mycobacterium species causing diffuse lepromatous leprosy. *Am. J. Clin. Pathol.* 130 856–864. 19019760 10.1309/AJCPP72FJZZRRVMM

[B20] Hernádez F. (2001). *The Mexican treasury: The Writings of Dr. Francisco Hernández*, ed. Varey S. Palo Alto, CA: Stanford University Press.

[B21] Hockings K. J. Mubemba B. Avanzi C. Pleh K. Düx A. Bersacola E. (2020). Leprosy in wild chimpanzees. *BioRxiv* [Preprint]. 10.1101/2020.11.10.374371 PMC8550970 34646009

[B22] Hofreiter M. Paijmans J. L. A. Goodchild H. Speller C. F. Barlow A. Fortes G. G. (2015). The future of ancient DNA: technical advances and conceptual shifts. *Bioessays* 37 284–293. 25413709 10.1002/bies.201400160

[B23] Honap T. P. Pfister L.-A. Housman G. Mills S. Tarara R. P. Suzuki K. (2018). Mycobacterium leprae genomes from naturally infected nonhuman primates. *PLoS Negl. Trop. Dis.* 12:e0006190. 10.1371/journal.pntd.0006190 29381722 PMC5790234

[B24] Inskip S. A. Taylor G. M. Zakrzewski S. R. Mays S. A. Pike A. W. G. Llewellyn G. (2015). Osteological, biomolecular and geochemical examination of an early anglo-saxon case of lepromatous leprosy. *PLoS One* 10:e0124282. 10.1371/journal.pone.0124282 25970602 PMC4430215

[B25] Jones B. A. Grace D. Kock R. Alonso S. Rushton J. Said M. Y. (2013). Zoonosis emergence linked to agricultural intensification and environmental change. *Proc. Natl. Acad. Sci. U.S.A.* 110 8399–8404. 23671097 10.1073/pnas.1208059110 PMC3666729

[B26] Karesh W. B. Dobson A. Lloyd-Smith J. O. Lubroth J. Dixon M. A. Bennett M. (2012). Ecology of zoonoses: natural and unnatural histories. *Lancet* 380 1936–1945. 23200502 10.1016/S0140-6736(12)61678-X PMC7138068

[B27] Köhler K. Marcsik A. Zádori P. Biro G. Szeniczey T. Fábián S. (2017). Possible cases of leprosy from the late copper age (3780-3650 cal BC) in Hungary. *PLoS One* 12:e0185966. 10.1371/journal.pone.0185966 29023477 PMC5638319

[B28] Krause-Kyora B. Nutsua M. Boehme L. Pierini F. Pedersen D. D. Kornell S.-C. (2018). Ancient DNA study reveals HLA susceptibility locus for leprosy in medieval Europeans. *Nat. Commun.* 9:1569. 10.1038/s41467-018-03857-x PMC5931558 29717136

[B29] Lahiri R. Krahenbuhl J. L. (2008). The role of free-living pathogenic amoeba in the transmission of leprosy: a proof of principle. *Lepr. Rev.* 79 401–409. 10.47276/lr.79.4.401 19274986

[B30] Martin J. (1986). *Treasure of the Land of Darkness: The Fur Trade and its Significance for Medieval Russia.* New York, NW: Cambridge University Press.

[B31] Mendum T. A. Schuenemann V. J. Roffey S. Taylor G. M. Wu H. Singh P. (2014). Mycobacterium leprae genomes from a British medieval leprosy hospital: towards understanding an ancient epidemic. *BMC Genomics* 15:270. 10.1186/1471-2164-15-270 24708363 PMC4234520

[B32] Minnikin D. E. Besra G. S. Lee O.-C. Spigelman M. Donoghue H. D. (2011). “The interplay of DNA and lipid biomarkers in the detection of tuberculosis and leprosy in mummies and other skeletal remains,” in Yearbook of Mummy Studies, 6 eds Gill-Frerking H. Rosendahl W. Zink A. Piombino-Mascasli D. (München: Verlag Dr. Friedrich Pfeil).

[B33] Monot M. Honoré N. Garnier T. Araoz R. Coppée J.-Y. Lacroix C. (2005). On the origin of leprosy. *Science* 308 1040–1042. 10.1126/science/1109759 15894530

[B34] Morozova I. Kasianov A. Bruskin S. Neukamm J. Molak M. Batieva E. (2020). New ancient Eastern European Yersinia pestis genomes illuminate the dispersal of plague in Europe. *Philos. Trans. R. Soc. Lond. B Biol. Sci.* 375:20190569. 10.1098/rstb.2019.0569 33012225 PMC7702796

[B35] Neukamm J. Pfrengle S. Molak M. Seitz A. Francken M. Eppenberger P. (2020). 2000-year-old pathogen genomes reconstructed from metagenomic analysis of Egyptian mummified individuals. *BMC Biol.* 18:108. 10.1186/s12915-020-00839-8 32859198 PMC7456089

[B36] Newton S. M. (1999). *Fashion in the Age of the Black Prince: A Study of the Years 1340-1365.* Suffolk: Boydell & Brewer.

[B37] O’Connor T. P. O’Connor T. (2008). *The Archaeology of Animal Bones.* College Station, TX: Texas A&M University Press.

[B38] Oliveira I. V. P. de M. Deps P. D. Antunes J. M. A. de P. (2019). Armadillos and leprosy: from infection to biological model. *Rev. Inst. Med. Trop. Sao Paulo* 61:e44. 10.1590/S1678-9946201961044 PMC6746198 31531622

[B39] Pavlopoulos G. A. Soldatos T. G. Barbosa-Silva A. Schneider R. (2010). A reference guide for tree analysis and visualization. *BioData Min.* 3:1. 10.1186/1756-0381-3-1 PMC2844399 20175922

[B40] Ploemacher T. Faber W. R. Menke H. Rutten V. Pieters T. (2020). Reservoirs and transmission routes of leprosy; a systematic review. *PLoS Negl. Trop. Dis.* 14:e0008276. 10.1371/journal.pntd.0008276 32339201 PMC7205316

[B41] Reiss R. A. (2006). Ancient DNA from ice age insects: proceed with caution. *Quat. Sci. Rev.* 25 1877–1893. 10.1016/j.quascirev.2006.01.009

[B42] Robbins G. Tripathy V. M. Misra V. N. Mohanty R. K. Shinde V. S. Gray K. M. (2009). Ancient skeletal evidence for leprosy in India (2000 BC). *PLoS One* 4:e5669. 10.1371/journal.pone.0005669 19479078 PMC2682583

[B43] Roberts C. A. (2020). *Leprosy: Past and Present.* Gainesville, FL: University of Florida Press.

[B44] Roberts C. A. Buikstra J. E. (2019). “Chapter 11–Bacterial Infections,” in *Ortner’s Identification of Pathological Conditions in Human Skeletal Remains*, 3rd Edn, ed. Buikstra J. E. (San Diego, CA: Academic Press), 321–439.

[B45] Roffey S. Tucker K. Filipek-Ogden K. Montgomery J. Cameron J. O’Connell T. (2017). investigation of a medieval pilgrim burial excavated from the leprosarium of St Mary magdalen winchester. UK. *PLoS Negl. Trop. Dis.* 11:e0005186. 10.1371/journal.pntd.0005186 28125649 PMC5268360

[B46] Schuenemann V. J. Avanzi C. Krause-Kyora B. Seitz A. Herbig A. Inskip S. (2018). Ancient genomes reveal a high diversity of Mycobacterium leprae in medieval Europe. *PLoS Pathog.* 14:e1006997. 10.1371/journal.ppat.1006997 29746563 PMC5944922

[B47] Schuenemann V. J. Singh P. Mendum T. A. Krause-Kyora B. Jäger G. Bos K. I. (2013). Genome-wide comparison of medieval and modern *Mycobacterium leprae*. *Science* 341 179–183. 23765279 10.1126/science.1238286

[B48] Scollard D. M. (2016). “Pathologenesis and pathology of leprosy,” in *International Textbook of Leprosy*, eds Scollard D. M. Gillis T. P.. Available online at: www.internationaltextbookofleprosy.org (accessed September 13, 2021).

[B49] Sharma R. Lahiri R. Scollard D. M. Pena M. Williams D. L. Adams L. B. (2013). The armadillo: a model for the neuropathy of leprosy and potentially other neurodegenerative diseases. *Dis. Model. Mech.* 6 19–24. 23223615 10.1242/dmm.010215 PMC3529335

[B50] Sharma R. Singh P. Loughry W. J. Lockhart J. M. Inman W. B. Duthie M. S. (2015). Zoonotic leprosy in the Southeastern United States. *Emerg. Infect. Dis.* 21 2127–2134. 26583204 10.3201/eid2112.150501 PMC4672434

[B51] Sharp P. M. Hahn B. H. (2011). Origins of HIV and the AIDS pandemic. *Cold Spring Harb. Perspect. Med.* 1:a006841. 10.1101/cshperspect.a006841 PMC3234451 22229120

[B52] Singh P. Benjak A. Schuenemann V. J. Herbig A. Avanzi C. Busso P. (2015). Insight into the evolution and origin of leprosy bacilli from the genome sequence of *Mycobacterium lepromatosis*. *Proc. Natl. Acad. Sci. U.S.A.* 112 4459–4464. 10.1073/pnas.1421504112 25831531 PMC4394283

[B53] Thomas R. (2019). “Nonhuman Animal Paleopathology—Are We so Different?,” in *Ortner’s Identification of Pathological Conditions in Human Skeletal Remains*, ed. Buikstra J. E. (Cambridge: Academic Press), 809–822. 10.1016/b978-0-12-809738-0.00023-5

[B54] Touati F.-O. (2021). “Lepers and leprosy: connections between east and west in the middle ages,” in *Leprosy and Identity in the Middle Ages*, eds Brenner E. Touati F.-O. (Manchester: Manchester University Press), 45–66.

[B55] Truman R. W. Singh P. Sharma R. Busso P. Rougemont J. Paniz-Mondolfi A. (2011). Probable zoonotic leprosy in the southern United States. *N. Engl. J. Med.* 364 1626–1633. 10.1056/nejmoa1010536 21524213 PMC3138484

[B56] Veale E. M. (1966). *The English Fur Trade in the Later Middle Ages.* New York, NY: Oxford University Press.

[B57] Walker-Meikle K. (2012). *Medieval Pets.* Suffolk: Boydell Press.

[B58] Wheat W. H. Casali A. L. Thomas V. Spencer J. S. Lahiri R. Williams D. L. (2014). Long-term survival and virulence of Mycobacterium leprae in amoebal cysts. *PLoS Negl. Trop. Dis.* 8:e3405. 10.1371/journal.pntd.0003405 25521850 PMC4270725

[B59] WHO (2020). Global leprosy (Hansen disease) update, 2019: time to step-up prevention initiatives–Situation de la lèpre (maladie de Hansen) dans le monde, 2019: le moment est venu d’intensifier les initiatives de prévention. *Wkly. Epidemiol. Rec.* 95 417–438.

